# The importance of developing an integrated data-driven modelling platform for herbicide resistance research: A review

**DOI:** 10.1016/j.heliyon.2025.e42564

**Published:** 2025-02-12

**Authors:** Md. Monirul Islam, Marta Monjardino

**Affiliations:** a Agriculture and Food, CSIRO, Adelaide, SA, Australia; b Department of Agricultural Economics, Bangladesh Agricultural University, Mymensingh-2202, Bangladesh

**Keywords:** Integrated data-driven modelling, Herbicide resistance, Sustainable agriculture, Integrated weed management, Potential drivers

## Abstract

Herbicide-resistant weeds pose a global challenge, constraining agricultural practices worldwide. Despite efforts to establish an integrated, data-driven framework, understanding the varied risks of herbicide resistance (HR) across different agroecological zones remains elusive. This review paper advocates for an integrated approach that incorporates socioeconomic, environmental, adoption behavior, and physiological factors to uncover insights into HR drivers and develop tailored management strategies. HR not only escalates production costs but also necessitates alternative weed management tactics, highlighting the urgency for proactive environmental management to mitigate soil health degradation and biodiversity loss. While current initiatives prioritize integrated weed management (IWM) like crop rotation and herbicide mixtures, challenges persist in integrating socioeconomic factors into predictive models and promoting the universal adoption of sustainable practices. Advancements in big data analytics, spatial modeling, and remote sensing offer promising avenues for predicting and managing HR across landscapes. This study proposes a research framework to predict the emergence and management of HR in agri-food systems. Additionally, the study utilizes a novel text-mining technique to conduct a comprehensive literature review, highlighting gaps in the development of data-driven modeling platforms for predicting HR emergence. The text mining findings explored that while common terms like weeds, resistance, herbicides, crops, management, and control are prevalent, research often lacks focus on predictive data-driven approaches for HR. Therefore, urgent development of an integrated national-scale approach to predict HR emergence is imperative. Global cooperation is essential for sharing best practices, data, and responses to emerging resistance threats.

## 1 Introduction

The Food and Agriculture Organization (FAO) reports that pests cause significant global crop losses, with up to 40 % of annual production affected, amounting to approximately $220 billion from plant diseases and $70 billion from invasive insects each year [1]. These losses underscore the urgent need for sustainable pest management strategies, particularly as pests, including weeds, develop resistance to synthetic herbicides [[2], [3], [4]]. The future use of pesticides faces several challenges, such as evolving resistance, regulatory constraints, public pressure to reduce pesticide use, and a slow pipeline for new crop protection products [2,5,6]. Resistance to synthetic herbicides by weeds threatens sustainable food and fiber production systems, as demonstrated by the increasing prevalence of herbicide resistance (HR) weeds globally [2,3].

Efforts to address these resistance challenges have largely focused on improving crop management practices. These include using weed-competitive crop cultivars, employing effective weed seed cleaning methods, adopting precision agriculture, and applying herbicides with diverse modes of action (MoA) to delay resistance development [7]. Research has further emphasized the phenotypic, physiological, and genetic underpinnings of HR, contributing to a deeper understanding of how resistance develops and persists [8,9]. However, these management strategies often fall short due to their narrow focus on field-specific resistance without considering broader spatial and temporal scales.

The integration of socioeconomic factors into resistance management has been recognized as a critical gap in current approaches. Historically, resistance management has been treated primarily as an ecological issue, overlooking the economic drivers that influence human decision-making in pest management practices [10]. This oversight limits the effectiveness of management strategies, particularly as resistance often involves complex interactions between ecological and economic systems. A transdisciplinary approach, combining spatial agroecological and socioeconomic factors with technology-driven pest management science, is essential [[11], [12], [13]].

Using integrated control practices has proven effective in reducing chemical use, costs, and the spread of resistance [14]. However, resistance management programs often fail to account for the spatial and temporal dimensions of resistance evolution, limiting their applicability across larger agricultural systems [15]. Many existing models for resistance prediction focus on limited practice inputs, typically at single-field scales, making them less useful for broader applications [16,17]. These models often rely on reactive, farmer-driven resistance detection methods, which may overlook early warning signs of resistance emergence and result in delayed or suboptimal management decisions.

The need for spatially explicit models that integrate socioeconomic and ecological factors becomes evident when considering the influence of human activities in agroecosystems. For instance, in Australia, while national resistance evolution data is collected at the field scale, it remains underutilized without transdisciplinary collaboration [18,19]. By integrating resistance data with environmental, climatic, and management variables, spatial predictive models can identify high-risk zones and forecast resistance hotspots, enabling targeted interventions that improve the efficacy of management practices [16,17]. Furthermore, incorporating resistance data into precision agriculture tools such as GPS-guided systems and remote sensing can facilitate site-specific herbicide applications, reducing selection pressure and enhancing the adoption of integrated weed management (IWM) strategies [20]. Despite advancements in IWM, existing research has yet to fully integrate these diverse factors into predictive models. Current methods often fail to account for the spatial variability of HR risks across different agroecological zones, which hinders the development of tailored management strategies.

Therefore, the development of an integrated, data-driven modeling framework is decisive in identifying high-risk zones, forecasting resistance hotspots, and enhancing the adoption of sustainable, region-specific management strategies. This review contributes to existing literature by addressing the critical gaps in current resistance prediction models. Most models focus on field-level or population-specific data, neglecting the spatial complexities of resistance and failing to incorporate socioeconomic drivers that influence management decisions [10,21,22].This transdisciplinary approach allows for a comprehensive understanding of resistance development, helping identify integrated management solutions that are scalable and applicable across different regions and farming systems. Moreover, the rise of big data analytics, spatial modeling, and remote sensing technologies presents a unique opportunity to address these limitations. By harnessing large-scale, integrated data sources, it is possible to create predictive models capable of mapping HR risks at a national or even global scale. The research outcomes aim to inform industry strategies, government policies, public opinions, and international stakeholders regarding the potential economic consequences of resistance development across different regions and systems. The overarching objective of this review is to develop a data-driven integrated modeling framework that proactively identifies resistance risks and effective management solutions, which industry stakeholders can implement in diverse agroecological and socioeconomic contexts.

In particular, this research aims to: (i) investigate the current occurrence and state of HR weeds in Australia and globally, and (ii) propose a conceptual framework that identifies research windows for predicting resistance emergence and effective management at relevant scales. This framework will support the adoption of sustainable strategies, drive industry innovation, and enhance the resilience of agri-food systems.

## 2 Scenario of the occurrence of HR weeds in Australia and the global context

**Fig. 1:**
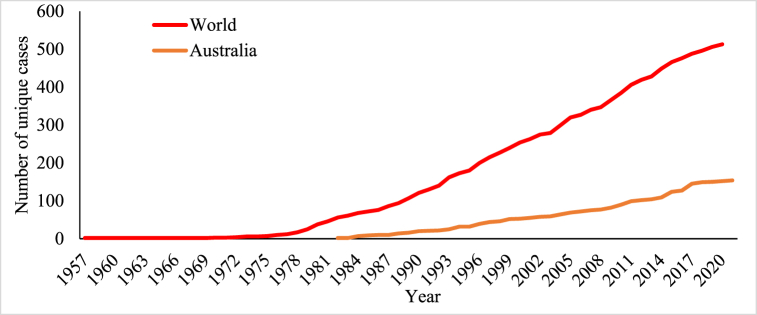
Chronological increase in unique resistant cases in Australia and the global context.

The international survey of HR weeds [23] tracks the occurrence and impact of HR weeds globally, providing essential data for this study. As of 2021, it has documented 518 distinct cases of HR worldwide, involving 267 species (154 dicots and 113 monocots) reported from 1957 to 2020. In comparison, Australia reported around 154 unique cases between 1982 and 2021 (Fig. 1).

**Fig. 2:**
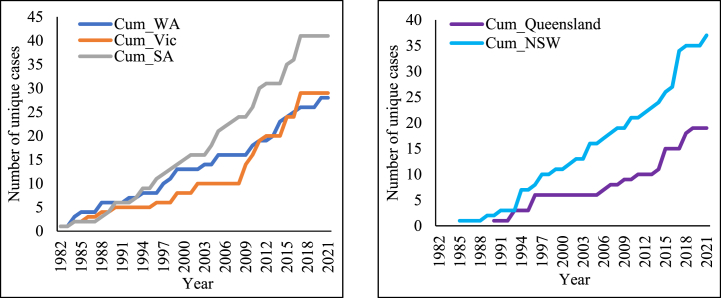
Chronological increase in unique resistant cases in different states in Australia.

Source: [23].

Source [23]:

In the early 2000s, HR weed populations proliferated widely across all of Australia's cropping regions, cementing its global leadership in HR. Before this period, Australian farmers had widely adopted conservation cropping techniques such as no-till and stubble retention [24]. While HR weeds have emerged in various parts of the world, Australia's grain-producing areas have witnessed particularly extensive development of resistance [23]. State-specific data for Australia is presented in Fig. 2, showing the chronological increase in unique resistant cases across different regions. South Australia (SA) reported the highest prevalence with 41 cases, while Queensland (QLD) had a lower prevalence of 19 cases.

This phenomenon is observed not only in herbicides but also in various organisms, including bacteria developing resistance to antibiotics, fungi to fungicides, insects to insecticides, and plants to herbicides. HR poses a significant threat to the long-term sustainability of grain production, resulting in lower crop yields and higher costs for weed management. In Australia, broadacre cropping systems heavily rely on continuous cropping and no-till practices that depend on herbicides for effective weed control [24].

Among Australian agricultural fields, HR is widespread among annual ryegrass populations, with resistance levels varying across regions and species. Other grass weeds like wild oats, brome grass, and barley grass are less common than annual ryegrass and tend to be linked to specific rainfall patterns or agronomic factors. Consequently, the emergence of HR weed populations has become a regular occurrence in Australia, impacting major crop weeds such as Lolium rigidum (annual ryegrass), Raphanus raphanistrum (wild radish), Avena spp. (wild oat), Bromus spp. (brome grass), and Hordeum spp. (barley grass) [[25], [26], [27], [28], [29], [30]]. These species have developed resistance to herbicides that target ACCase (HRAC Group A) and ALS enzymes (HRAC Group B), reducing the effectiveness of commonly used herbicides [31].

## 3 Herbicide resistance: global trends, economic impacts, and key drivers

### 3.1 Worldwide occurrence of HR weeds

**Fig. 3:**
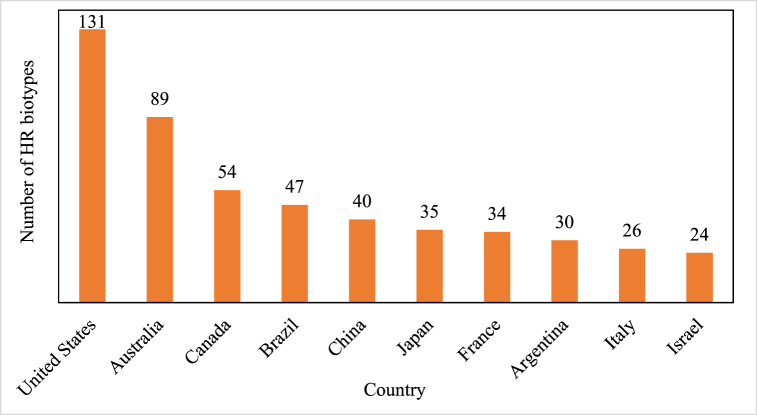
The number of HR biotypes for the top 10 countries.

Source: [23].

Fig. 3 highlights the number of HR biotypes reported in the top 10 countries globally. The United States (US) leads with 131 biotypes, followed by Australia with 89 biotypes. Canada and Brazil rank third and fourth with 54 and 47 biotypes, respectively. China, Japan, and France show a relatively moderate number of resistant biotypes, ranging between 40 and 34, while Argentina, Italy, and Israel report the lowest numbers in this group, ranging from 30 to 24 biotypes. The data underscores significant regional variations in HR, with the US and Australia showing markedly higher numbers. These disparities may reflect differences in herbicide usage intensity, cropping systems, resistance management practices, and reporting efforts (Fig. 3).

### 3.2 HR patterns by crop and weed families

**Fig. 4:**
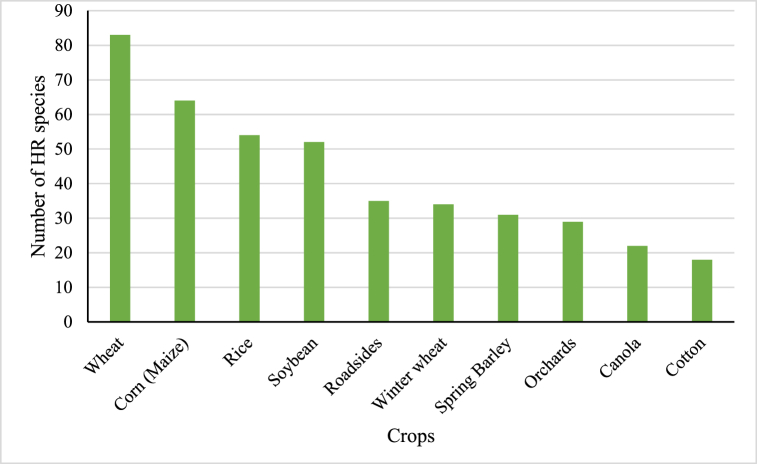
Number of HR species by crop (top 10).

Source: [23].

Fig. 4 displays the number of weed species that have acquired resistance in various agricultural and non-agricultural environments. Wheat and corn lead with 83 and 64 resistant weed species, respectively, followed by rice (54) and soybean (52). This is consistent with expectations, as these crops are widely treated with herbicides across extensive areas and over extended durations. Roadsides (35 species) and winter wheat (34 species) closely follow, where resistance develops due to frequent and repeated herbicide applications (often exceeding five times per season) required to control vegetation.

**Fig. 5:**
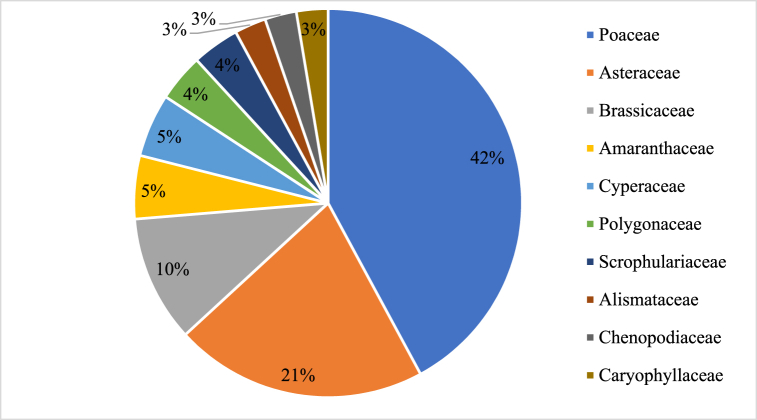
Percentage of HR species by weed families (top 10).

Source: [23].

Fig. 5 shows the percentage of HR species by weed families. Poaceae and Asteraceae have occupied 42 % and 21 % of HR species among weed families, respectively, followed by Brassicaceae (10 %) and Amaranthaceae (5 %).

### 3.3 Economics of HR management

Weeds pose a significant threat to agricultural production, and herbicides have been the primary tool for weed control for over five decades [32]. However, agriculture faces a pressing issue with HR, which has negative impacts on crop yields and financial outcomes [33]. The emergence and spread of HR weeds have led to increased costs, decreased yields, and the adoption of alternative weed management strategies. However, IWM practices provide a solution to mitigate or delay the development of HR and enhance profitability [34]. Best management practices (BMPs) encompass mechanical, biological, chemical, cultural, and physical control methods aimed at sustainable weed management. BMPs include practices such as crop rotation, herbicide rotation, following recommended application rates and schedules, regular field monitoring, and sanitation [15].

Research by the University of Arizona and the US Department of Agriculture challenges traditional approaches to resistance management. They assessed the effectiveness of resistance management strategies based on their impact timeframe. Economic modeling evaluated the profitability of managing glyphosate resistance in Canada fleabane through rotations of corn, soybean, and corn-soybean. The results show that while resistance management initially reduces profits, it enhances profitability over an 18-year period. The results demonstrate that while resistance management strategies may initially reduce profits due to the costs associated with adopting new practices, such as the use of alternative herbicides, diversified weed control methods, or investment in precision agriculture technologies, these strategies tend to enhance profitability over the longer term [35]. However, a key challenge arises from the practical reality that farmers often prioritize immediate profitability, particularly in regions facing economic pressures or limited access to capital. The prospect of reduced short-term profits may deter some farmers from adopting resistance management strategies, as they may lack the financial capacity or the patience to endure temporary losses in favor of potential long-term gains. By the second year, the benefits of resistance management outweigh its costs in all three cropping systems. Managing resistance over 20 years resulted in profit gains of $158 per hectare ($64 per acre) for corn, $137 per hectare ($55 per acre) for corn-soybean rotations, and $55 per hectare ($22 per acre) for soybean. These profit increases represent a 14–17 % rise for growers over two decades. Further research is necessary to explore how these findings apply across different cropping systems and regions [36].

According to Llewellyn et al. [37], weeds cost Australian grain growers a total of $3318 million, which includes yield losses of 2.76 million tonnes. Table 1 outlines the top five weeds that drive up herbicide costs for Australian growers in winter cereal crops. In their 2016 study, Llewellyn et al. [37] calculated the expenses related to HR weeds based on the extent of resistance and the additional herbicides required to control them. It includes the cost of yield loss due to in-crop and fallow weeds and grain contamination costs as well as weed control costs such as herbicide and nonherbicide practices. These findings are based only on winter crop production and did not include fallow weed management or weed management practices other than selective herbicides and cultivation.

**Table 1:** Top 5 HR weeds in terms of management costs.

Weeds	Management cost (million)
Ryegrass	$103.2
Wild radish	$19.7
Wild turnip	$7.8
Wild oats	$6.2
Barnyard grass	$4.1

The challenge of HR in weed management is influenced directly by how effective and prevalent herbicides are, which impacts the speed at which resistance develops [38]. Economic factors are critical in shaping farmers' choices regarding resistance management practices, as evidenced by Riar et al. [39], who studied cotton and soybean crops and found that farmers adopted thirteen out of sixteen recommended practices primarily due to cost considerations.

**Fig. 6:**
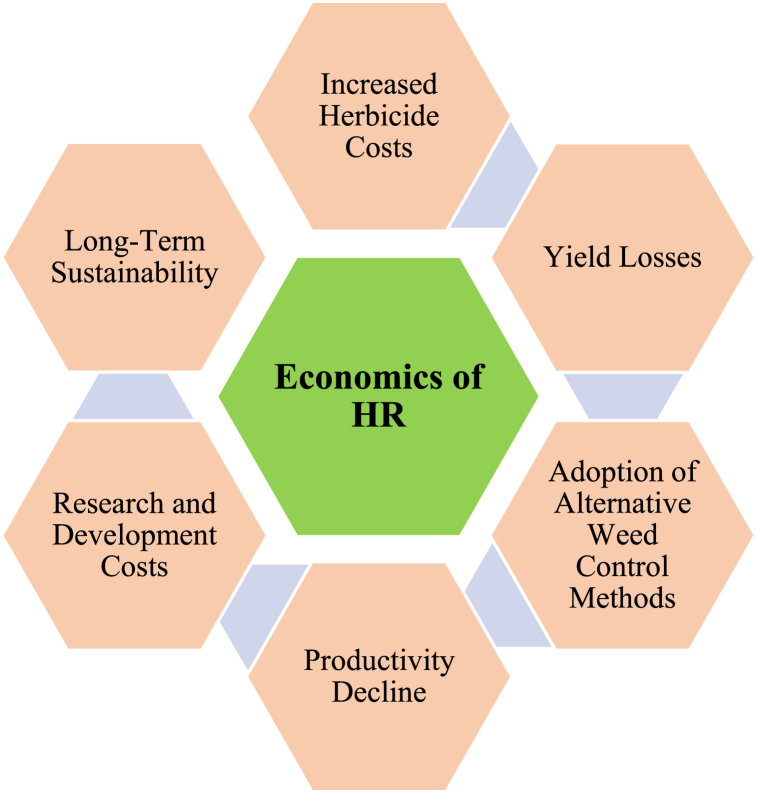
Factors determining the economics of HR for the farmer.

To grasp the economic implications of HR, factors like regional variations, crop types, and specific weed species must be taken into account. The degree of resistance, effectiveness of alternative control methods, and the adoption rates of management practices all significantly influence the economic outcomes (see Fig. 6). These factors highlight the importance of proactive management strategies and economic evaluations in effectively addressing HR.

HR often compels farmers to adopt alternative herbicide formulations or mixtures, which can lead to increased herbicide costs. However, this approach may not always align with the recommendations of the Herbicide Resistance Action Committee (HRAC), which advocates for strategies that prioritize the use of herbicides with different MoA to effectively manage resistance. HRAC promotes diversified herbicide rotations and tank mixes that combine multiple active ingredients from different MoA groups to reduce resistance development. For example, tank mixing Group A (Fop-Dim) herbicides with Group B (Dinitroaniline) herbicides, such as pendimethalin or diflufenican, can enhance control and minimize resistance risks in weed species like ryegrass.

HR weeds compete with crops for resources, leading to yield losses that impact farm income. To mitigate resistance, farmers are increasingly adopting non-chemical methods such as mechanical cultivation, crop rotation, cover cropping, and manual weeding [15,34,39]. Although these practices are often more labor- and cost-intensive than herbicide-based methods, they help reduce overall production expenses [20]. Resistance decreases crop productivity and necessitates substantial investments in the development of new herbicides or herbicide-tolerant crop varieties, further increasing input costs [40].

The adoption of herbicide-tolerant crops, particularly glyphosate-tolerant varieties, has exacerbated resistance issues in some regions, such as the US, where glyphosate-resistant weed populations, such as Amaranthus palmeri (Palmer amaranth) and Conyza spp. (horseweed), have emerged. The over-reliance on glyphosate has reduced its effectiveness, leading to increased use of alternative herbicides or costly tank mixes [19]. While herbicide-tolerant crops can be integrated into IWM strategies, their use in isolation has frequently contributed to resistance evolution. Thus, context-specific resistance management strategies are needed to balance economic and ecological sustainability, emphasizing HRAC-recommended practices to slow resistance evolution and maintain the long-term efficacy of herbicide use.

### 3.4 Importance of the prediction of HR

**Fig. 7:**
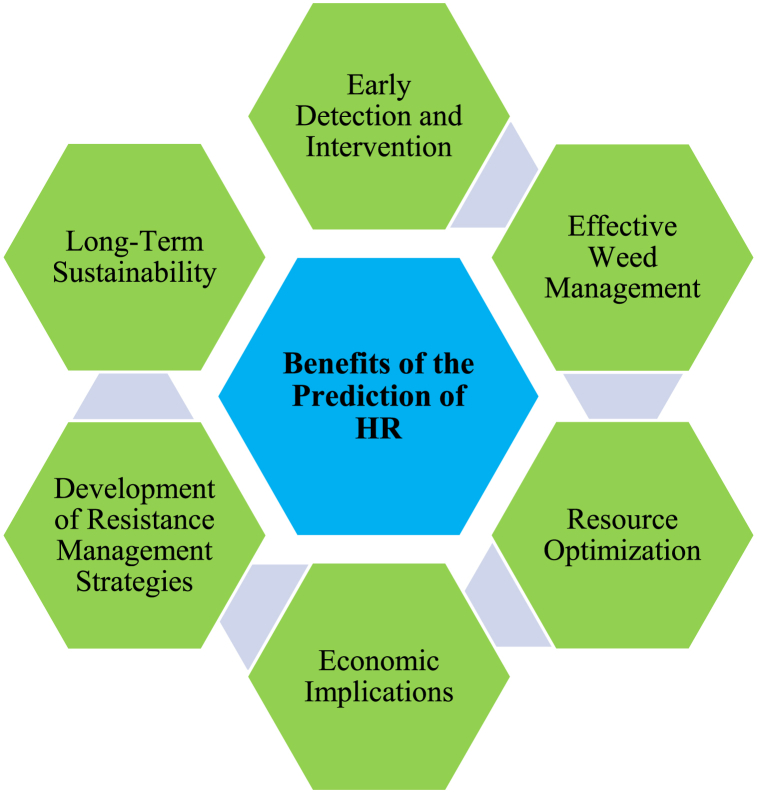
Factors involved in the benefits of the prediction of HR for the farmers.

Research into predicting HR is critical for sustainable agriculture and effective weed management [19]. Early detection through predictive research allows farmers and stakeholders to identify HR weed populations promptly, enabling timely intervention and reducing their impact on crop production. Additionally, predicting resistance helps in the development of more efficient weed management strategies, including diverse herbicide rotations, IWM approaches, and region-specific control methods tailored to different resistance risks and weed species [41]. This proactive management contributes to the sustainability of weed control systems by preserving the effectiveness of herbicides (see Fig. 7). Predictive research optimizes the use of resources for weed control, allowing farmers to apply herbicides strategically and reduce reliance on single herbicides or MoA, thereby minimizing costs and environmental impacts [15,42]. Effective resistance prediction helps farmers make informed decisions to mitigate yield losses and economic risks associated with resistance [19]. Moreover, it supports investors in selecting cost-effective weed management tools, enhancing profitability [20,43].

Research on HR prediction also provides essential insights for developing regional and national strategies to manage resistance. This knowledge supports policymakers, researchers, and industry stakeholders in implementing targeted educational and regulatory measures to combat HR and promote sustainable weed control practices. By understanding how resistance evolves, farmers can adopt proactive weed management strategies that reduce their dependence on herbicides while ensuring their long-term efficacy. This approach enhances the productivity and profitability of agricultural systems while minimizing the environmental footprint of herbicide use [44].

In summary, research on HR prediction is crucial for early detection, efficient weed management, resource optimization, economic stability, the development of effective resistance management strategies, and the long-term sustainability of agriculture. Integrating this research into practical weed management approaches empowers farmers and agricultural stakeholders to make informed decisions, effectively manage HR, and sustain productive and environmentally friendly cropping systems.

### 3.5 Major drivers of HR

To combat HR globally, a comprehensive strategy integrating various interventions is essential. IWM practices play a pivotal role in reducing dependency on herbicides and alleviating selection pressure on resistant weed populations. Rotating herbicides with different MoA and using herbicide mixtures disrupts the survival and reproduction of resistant weeds by reducing selection pressure. Effective herbicide stewardship, including adherence to label instructions, proper dosage, timing, and regular resistance monitoring, is critical to early detection and prevention of resistance. Educational programs for farmers and agronomists are key to promoting better resistance management practices.

**Fig. 8:**
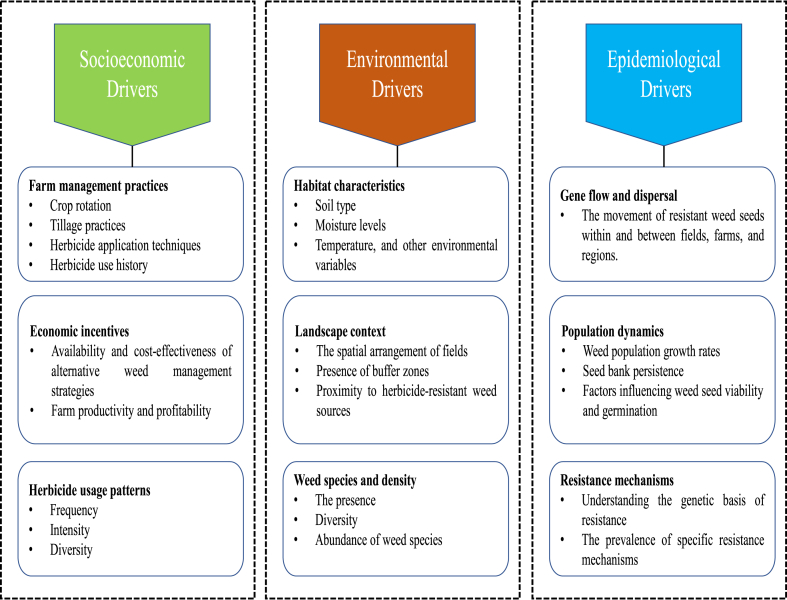
Major drivers, along with their subcomponents.

Reducing resistant weed prevalence requires adopting diverse crop rotations and integrating multiple crops to support alternative control methods like cultural practices and crop competition. Herbicide-tolerant crop varieties can also aid in managing resistant weeds, though their responsible use and stewardship are necessary to prevent rapid resistance evolution. Addressing HR globally necessitates international collaboration to share information, best practices, and experiences, facilitated by governments and regulatory bodies through policies promoting sustainable weed management, such as herbicide restrictions and support for research and extension services (Fig. 8).

Adapting HR management strategies to local and regional contexts, considering variables such as weed species, crop systems, farming practices, and available resources, is crucial. A successful global approach to HR management must be comprehensive, adaptable, and tailored to specific local conditions. Predicting HR requires an interdisciplinary approach that integrates socioeconomic, environmental, and epidemiological factors, as these dimensions provide critical insights into the development and spread of resistance [10].

Socioeconomic factors significantly influence farming practices, resistance management strategies, and herbicide usage patterns. These factors encompass the frequency, intensity, and types of herbicides employed in agricultural systems, collectively known as herbicide usage patterns. Farm management practices such as crop rotation, tillage methods, herbicide application techniques, and the educational background of farmers also play pivotal roles [5,45]. Economic indicators such as access to information, training opportunities, government policies, affordability of alternative weed management techniques, and regulatory frameworks further mold these practices. Analyzing historical data on herbicide use, farm management practices, and socioeconomic indicators can unveil patterns and correlations associated with the emergence of HR.

Environmental factors exert a significant influence on the dynamics of HR. Factors such as weed species diversity, density, and habitat characteristics—including soil type, temperature, rainfall, and other environmental variables—affect weed growth and the efficacy of herbicides [20,46]. Landscape context, including field placement, buffer zones, and the presence of HR weeds, also contributes to these dynamics. Gathering environmental data through methods like remote sensing, field studies, and geographic information systems (GIS) is crucial for modeling the dynamics of HR [16]. HR can be compared to an epidemiological process, where resistance genes mutate and spread within weed populations. Understanding the genetic basis of resistance and the prevalence of resistance mechanisms is essential. The movement of resistant weed seeds, termed gene flow and dispersal, within and between fields and regions influences the spread of resistance. Factors affecting weed population dynamics, such as growth rates, the persistence of seed banks, and conditions impacting seed viability and germination, also contribute to these processes [20,46]. Genetic data and epidemiological models are utilized to simulate and predict the spread of herbicide resistance over time.

Various modeling techniques, including statistical models, machine learning algorithms, and simulation models, leverage these parameters to predict HR [47]. These models are trained and validated using historical data on occurrences of HR and the associated socioeconomic, environmental, and epidemiological factors [17,48].

## 4 Literature review and new research window for sustainable HR research

After conducting a comprehensive review of numerous scientific papers and reports spanning several decades, it was found that most research on HR primarily focuses on experimental studies or literature reviews. Furthermore, the spatial scale component is often overlooked in these studies. Additionally, mechanistic models, commonly used in the industry, are limited in their application as they consider only a few practice inputs and are restricted to a single field or population. A crucial gap remains in effectively integrating socioeconomic data into predictive systems representing crop-weed management decisions. While econometric models are not commonly used for predicting HR in agricultural systems, they can provide valuable insights into HR's economic drivers and implications. Although these studies [20,49] discuss the adoption of IWM practices and the economic impacts of HR, they may not directly illustrate the prediction of HR using econometric models. However, they shed light on the importance of economic factors, decision-making processes, and policy considerations in managing HR in agricultural systems (Table 2).

**Table 2:** Summary of recent literature reviews on HR research.

S.N.	Authors	Literature name	Method/ Approach	Key findings and impact
Scientific articles				
1.	Vijayarajan et al. [50]	Grass-weed challenges, herbicide resistance status and weed control practices across crop establishment systems in Ireland's mild Atlantic climate.	Experimental with field survey	Continuous monitoring of grass-weed challenges, grower attributes, and IWM strategies is essential. Given complex resistance patterns, prompt adoption of effective IWM practices, such as non-inversion tillage, is crucial across agricultural systems.
2.	Height et al. [51]	Opportunities to manage herbicide resistance through area-wide management: lessons from Australian cropping regions.	Field survey	This research seeks support for area-wide herbicide resistance management, primarily through information sharing rather than coordination or joint activities.
3.	Gascuel-Odoux et al. [52]	A research agenda for scaling up agroecology in European countries	Review	This study focuses on constructing and disseminating a research agenda for agroecology, using an example from a major research institute.
4.	Jacquet et al. [53]	Pesticide-free agriculture as a new paradigm for research	Review work	Agricultural research is crucial for advancing innovative, pesticide-free approaches. This study identifies key research fronts to develop strategies for achieving pesticide-free agriculture.
5.	Hulme [5]	Global drivers of herbicide-resistant weed richness in major cereal crops worldwide	Macroecological approach	The worldwide occurrence of HR weeds is probably underestimated, particularly in countries with limited capacity for herbicide research.
6.	Busi et al. [54]	Herbicide resistance across the Australian continent	Experimental	The first study to report the frequency and geographical distribution of resistance in Lolium rigidum on a national scale
7.	Walsh and Ross [44]	Economic implications of the loss of glyphosate and paraquat on Australian mixed enterprise farms	Review paper	Banning glyphosate and paraquat in Australia alone will raise cropping costs, reduce farm profits, and shift farming systems towards sheep production, raising greenhouse gas emissions. A global ban on these herbicides would increase grain prices.
8.	Bitetto et al. [18]	A data-driven approach to measuring epidemiological susceptibility risk around the world	A data-driven approach, PCA, DFM	The results show that the robust principal component analysis (PCA) method accounts for about 90 % of total variability, while the dynamic factor model (DFM) accounts for about 76 %.
9.	Comont and Neve [19]	Adopting epidemiological approaches for herbicide resistance monitoring and management	Experimental and field survey	The discipline of epidemiology, which methodically investigates the scope, distribution, and causative factors of a detrimental organism or condition, can play a crucial role in comprehending the development, selection, and dissemination of HR.
10.	Somerville et al. [16]	Spatial modelling of within-field weed populations: A review	Static and spatiotemporal models	The research emphasizes the advantages and limitations of existing methods, along with present and upcoming areas of focus for employing spatial models. These areas encompass targeted weed treatments, economic analysis, IWM, HR, and more.
11.	Petit et al. [55]	Biodiversity-based options for arable weed management. A review	Review	This study provides evidence that biotic interactions can significantly impact weed growth at various life cycle stages. However, implementing biodiversity-based management systems that utilize ecosystem services for weed regulation remains challenging.
12.	Hicks et al. [10]	The factors driving evolved herbicide resistance at a national scale	Experimental research	The study identified some influential factors that evolved HR nationally in the United Kingdom (UK), including weed densities and economic costs.
13.	Mascanzoni et al. [45]	Epidemiology and agronomic predictors of herbicide resistance in rice at a large scale	Experiment (secondary data)	This is the first study that determines the degree of association between HR and a few important predictors at a large scale.
14.	Schütte et al. [11]	Herbicide resistance and biodiversity: Agronomic and environmental aspects of genetically modified herbicide-resistant plants	Reviews	The adoption of HR crops affects agronomy, agricultural practices, and weed management, leading to various consequences, including biodiversity loss.
15.	Evans et al. [42]	Managing the evolution of herbicide resistance	Experimental research	Achieving effective long-term weed management necessitates implementing genuinely diverse management practices that actively minimize the selection for HR traits.
16.	Gual and Norgaard [13],	Bridging ecological and social systems coevolution: A review and proposal	Review paper	(i) A general framework that accommodates advances in explaining sociocultural evolution in social sciences. (ii) Identifying the precise mechanisms that can establish connections between this knowledge and existing understanding in the biological sciences.
17.	Rick et al. [56]	Herbicide resistance in Rigid Ryegrass (Lolium rigidum) has not led to higher weed densities in Western Australian cropping fields.	Experimental	Growers are generally maintaining low densities in fields with herbicide-resistant rigid ryegrass. The most common rigid ryegrass density at harvest time was less than one plant m22 in resistant and susceptible populations.
18.	Noailly 2008 [12]	Coevolution of economic and ecological systems	Economy–environment coevolution model	(i) A large pest population reduces economic revenues. (ii) economic activities select for resistant genes, and (iii) the spread of resistant genes affects the size of the pest population.
19.	Duke and Stephen [57]	Glyphosate: a once-in-a-century herbicide	Mini review	Glyphosate/glyphosate-resistant crop weed management offers significant environmental and other benefits over the technologies it replaces.
20.	Ghersa et al. [58]	Coevolution of agroecosystems and weed management	Review	Weed management practices have become closely linked to social and economic, rather than biological, factors.
**Reports**				
R1.	Ross McLeod [59]	Annual costs of weeds in Australia, 2018	Report: Economic surplus approach	Overall costs have increased by more than 20 % over the 14 years since the Sinden et al. [60] study. Using the ' economic surplus ' approach, an average production loss cost of $4823 million is estimated for winter and summer broad-acre cropping, rice, cotton, horticulture, and livestock industries.
R2.	Preston [61]	Section 1: Economic Benefits of integrated weed management	Report	(1) Weed seed carryover in the soil seed bank significantly impacts returns in future years. (2) Calculating returns over the long term (e.g., 10 years) will help determine the actual value of weed management options.
TABLE E2 Benefits of non-chemical weed control options (Gorddard et al., 1995) [62].				
TABLE E3 Net present values (NPV) of alternative weed control strategies in a 20-year continuous cropping (wheat–lupin) rotation in the central wheatbelt of Western Australia [63].				
R3.			RIM Model	A tool to evaluate the profitability of ryegrass (Lolium rigidum Gaud.) control methods in the no-till broadacre cropping systems of the Southern Australian grain belt, short- and long-term and at the paddock scale [[64], [65], [66]].

It is important to note that studies predicting HR often use biological and agronomic models rather than econometric ones. These models estimate the likelihood of HR emergence by incorporating factors such as weed biology, herbicide use patterns, genetics, and environmental conditions [67]. Examples of such models include the Weed Resistance Integrated Prediction (WRIP) model and the Herbicide Resistance Evolution Simulator (HERMES) [17].

Current literature emphasizes the need for localized solutions that address HR risks but lacks a comprehensive framework that integrates spatial modeling, remote sensing, and big data analytics to anticipate HR emergence. Research often neglects the role of socio-economic factors such as farmer adoption behavior, economic pressures, and regional disparities in HR spread. Without integrating these variables, predictive models tend to overlook the complex interactions that drive HR development. The absence of a unified, data-driven platform capable of synthesizing diverse datasets—ranging from agronomic practices to socioeconomic factors—poses a significant barrier to effectively addressing HR challenges. A more integrated approach that draws on data-driven technologies, shared global knowledge, and collaborative efforts is essential to develop sustainable management strategies for HR at the landscape level. This research gap emphasizes the urgent need for coordinated national and international efforts to advance predictive models and support sustainable agricultural practices that ensure food security and mitigate environmental degradation.

## 5 Text mining: uncovering research gaps in existing HR literature

**Fig. 9:**
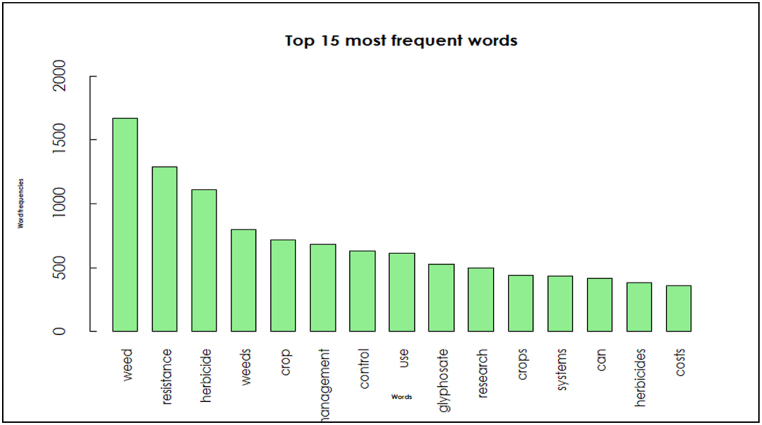
The most frequent words in HR research.

In recent times, vast amounts of text flow through the internet from digital libraries, repositories, emails, blogs, and social media accounts [68]. Extracting valuable knowledge from these data patterns and trends requires significant effort, given their sheer volume [69]. Text mining offers a robust solution for analyzing these textual sources to uncover meaningful patterns and insights [70,71], making it widely applicable across academia, web applications, the internet, and various industries [72]. The word cloud diagram emphasizes that research into developing an integrated data-driven modeling platform remains relatively sparse. Most studies focusing on themes like weed, resistance, herbicide, crop, management, and control are prominently featured in the bar diagram, which displays the top 15 most frequent words (Fig. 9).

The word cloud displays the 100 most frequently used words from a dataset comprising 20 research articles and two reports on global HR. These insights are integral to research aimed at predicting and managing HR across different scales, providing valuable guidance for adopting sustainable strategies, fostering industry innovation, and bolstering the resilience of agricultural and food systems.

**Fig. 10:**
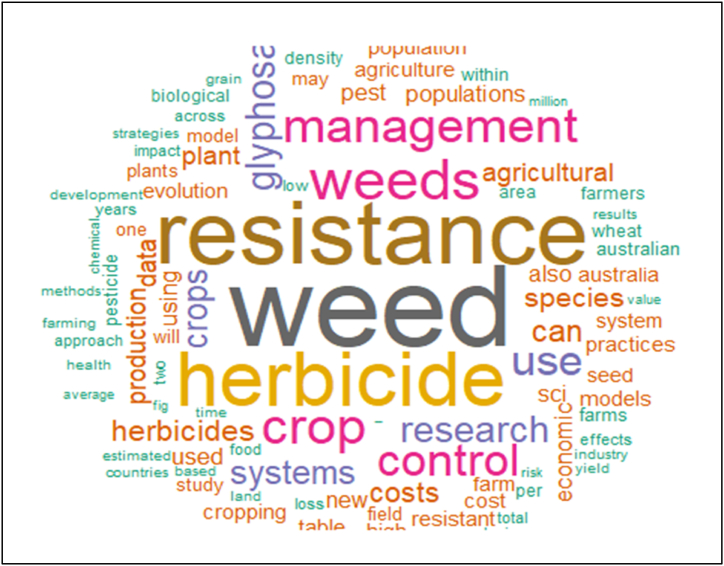
Word cloud result for the HR research.

Furthermore, the word cloud analysis highlights extensive coverage of review papers and reports that explore topics such as HR, management strategies, the economic impacts of resistance, coevolution within agroecosystems, weed control methods, epidemiology, and factors influencing HR (Fig. 10).

**Fig. 11:**
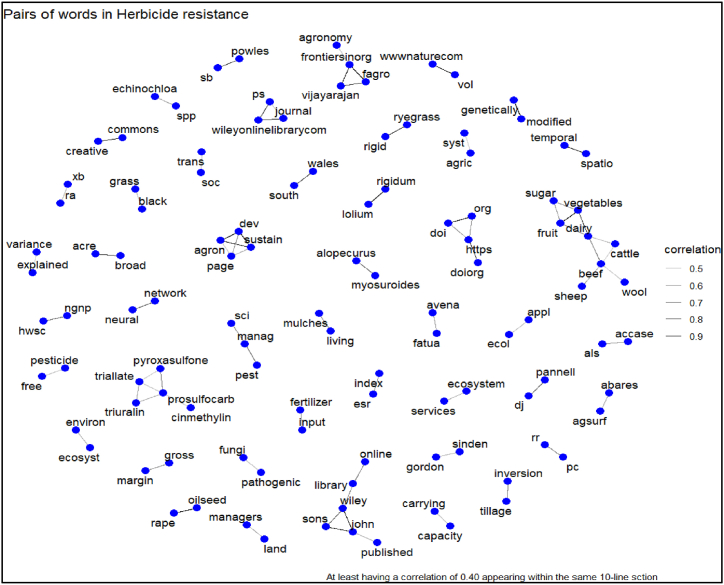
Network matrix for the HR research.

The correlations and co-occurrences show that the two words are more often used together in the same context. The higher the correlation, the greater the chances of using two words in the same context. We focused only on those topmost words and their associations to emphasize our most frequently used words in different HR research. For instance, words such as "pesticide-free", "genetically modified", "tillage-inversion", and "herbicide-resistance" were highly correlated (0.90). There was also a high correlation (0.80) between the words "gross-margin", "supply-demand", "black-grass", "rigidum-lolium", etc. (Fig. 11).

These co-occurrences suggest that HR research tends to be fragmented, with a predominant focus on specific concepts without fully integrating related aspects. By identifying such high correlations, this analysis underscores the necessity for a more holistic approach to HR research—one that integrates socioeconomic, environmental, and biological factors. For instance, understanding how socioeconomic drivers like gross margin impact HR spread, or how the co-occurrence of genetically modified crops and HR can shape resistance patterns, could lead to more comprehensive, data-driven predictive models.

## 6 Conceptual framework for an optimistic research window

This study aims to develop a data-driven model for predicting the emergence and management of HR in agricultural and food systems. This model will integrate datasets from various components of a data generation platform, including primary data, remotely sensed or captured data, and ancillary data. The primary datasets will be divided into field-level experimental data and management factors. The field-level experimental dataset will include information such as resistance status, weed abundance, cropping intensity, cropland area, crop type, and sowing season. The management factors dataset will include data on crop rotation, historical management practices, costs, adoption status, farmer perceptions/preferences, and consultant/agronomy advisor.

**Fig. 12:**
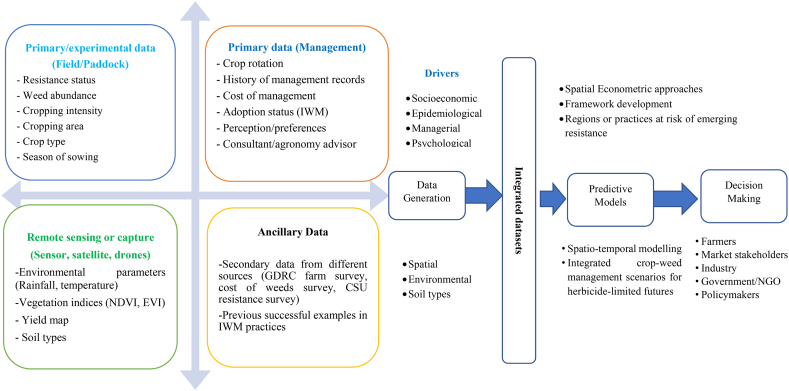
Proposed research framework to predict the emergence and management of HR in agri-food systems.

Incorporating remotely sensed or captured datasets will allow for the inclusion of multiple variables related to environmental and weather conditions (e.g., temperature, rainfall), vegetation health, yield estimates, and loss assessments across spatial and temporal scales. These diverse data sources will provide robust datasets to calibrate the model for agroecological considerations. The primary dataset generated through this research will be essential for estimating the economic impact of HR and assessing the environmental benefits of IWM practices. Additionally, remote sensing data will aid in identifying regions or agricultural practices vulnerable to resistance. The research aims to create a location-specific resistance map, to reduce the heavy reliance on herbicide applications in global agricultural and food systems (Fig. 12).

**Fig. 13:**
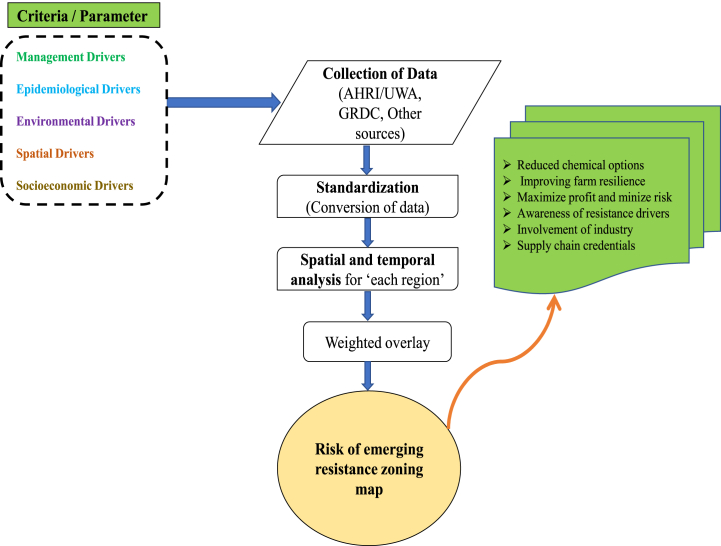
The process of exploring the regions at risk of emerging resistance to herbicides.

Once the framework is developed, the following steps will be necessary to put the model into operation. Various datasets on management, epidemiology, environmental, spatial, and socioeconomic factors will be collected from sources such as the Australian Herbicide Resistance Initiative (AHRI), the University of Western Australia (UWA), Grains Research and Development Corporation (GRDC), and other relevant organizations. The data will be sorted, standardized, and pre-processed to ensure consistency. Datasets will be categorized according to GRDC agroecological zones. Given the framework's consideration of multiple parameters, each parameter will be assigned appropriate weights for the final model implementation. A map depicting the risk of emerging HR will be generated, aiding in reducing reliance on chemical applications, enhancing farm resilience, and promoting sustainable IWM practices. Fig. 13 provides a comprehensive overview of the entire process for predicting regions at risk of emerging HR.

The risk of emerging resistance zoning map provides regional insights into HR risks by integrating key drivers such as management, environmental, epidemiological, spatial, and socioeconomic factors. While the map identifies high-risk and low-risk areas, its practical application to farm-level decision-making requires combining these regional insights with site-specific data. By incorporating farm-level factors such as weed species composition, cropping systems, herbicide usage history, and precision agriculture technologies (e.g., GIS and remote sensing), farmers can develop tailored IWM strategies. These strategies may include rotating herbicide MoA, diversifying control methods, and optimizing crop rotations to mitigate resistance risks effectively. Additionally, decision-support tools derived from the zoning map can provide actionable recommendations for herbicide application and resistance prevention. Collaboration among farmers, researchers, and industry stakeholders further enhances the approach, ensuring the zoning map evolves with field-level observations. This integration of regional risk assessments with farm-specific management practices offers a comprehensive and dynamic framework for addressing HR in a sustainable and targeted manner.

## 7 Unlocking potential with a data-driven approach

This review paper seeks to explore the importance of creating an innovative methodology to tackle the challenge of achieving sustainable crop protection amidst reducing chemical options. The proposed data-driven approach is anticipated to significantly influence both research and industry practices in managing resistance and pesticides. Unlike conventional methods that narrowly focus on individual population mechanics in HR prediction research, this new approach will adopt a broader socioeconomic perspective on a large scale. Current methods often face limitations and heavily rely on extrapolation to forecast resistance risks and guide industry decisions across different levels of the crop and crop protection supply chain.

The review predicts the development of a robust data-driven predictive capability, leveraging collective research in crop-weed systems, socioeconomics, and digital agriculture, alongside newly accessible national databases and advanced analytics. Starting from its initial development in Australia and in collaboration with international partners, this project aims to establish novel prediction methodologies applicable worldwide. By providing a reliable means to forecast resistance and its influencing factors on a large scale, the expected impact includes reducing the environmental footprint of pesticide use, enhancing trade opportunities along the value chain, supporting industry compliance with regulations, and identifying priorities for research, development, and extension investments.

## 8 Conclusion

In summary, despite the evolution and the epidemiology of HR primarily reflecting specific local management and mechanistic model's contexts, an integrated data-driven approach will examine robust and sensible insights into the drivers of HR from different angles of HR management strategies by incorporating socioeconomic, environmental, adoption behaviour, and physiological aspects. It is highly likely that the broader synthesis of the literature review of HR research currently represents the significance of the gap and an urgent need related to the development of a data-driven platform for different agroecological contexts for better management strategies. The economic impact of HR is substantial, leading to increased production costs, reduced yields, and the need for alternative weed management strategies. Current strategies predominantly focus on IWM practices, such as crop rotation and herbicide mixtures, to mitigate resistance. However, challenges remain in integrating socioeconomic factors into predictive models and ensuring the widespread adoption of sustainable practices. Advances in big data analytics, spatial modeling, and remote sensing offer promising avenues for developing proactive, data-driven frameworks to predict and manage HR. These technologies enable a more comprehensive understanding of resistance dynamics at landscape scales, aiding in targeted management strategies.

Research results and insights through this proposed framework will inform industry strategy, government policy, public perception, and international stakeholders about the potential economic implications across various regions/systems and what practices must be adopted at the farm level when essential herbicides are unavailable. Collaborative efforts among countries are essential to share best practices, data, and experiences in HR management. This includes establishing global networks for monitoring resistance patterns and coordinating responses to emerging resistance threats. Governments and private sectors should increase investment in research and innovation aimed at developing new herbicides with novel MoA and herbicide-tolerant crop varieties. By adopting proactive and sustainable strategies, global agriculture can mitigate the impact of HR weeds and ensure food security for future generations.

## CRediT authorship contribution statement

**Md Monirul Islam:** Writing – review & editing, Writing – original draft, Visualization, Validation, Software, Resources, Methodology, Investigation, Formal analysis, Data curation, Conceptualization, Supervision. **Marta Monjardino:** Writing – review & editing, Writing – original draft, Supervision, Project administration, Investigation, Funding acquisition, Conceptualization, Resources.

## Data availability statement

The data presented in this manuscript are available on request from the corresponding author.

## Funding

Research funded by 10.13039/501100000943CSIRO Winanga-y Postdoctoral Fellowship A&F: 17. Pesticide resistance in agricultural systems (OD-228894).

## Declaration of competing interest

The authors declare the following financial interests/personal relationships which may be considered as potential competing interests: Md. Monirul Islam reports financial support, administrative support, and statistical analysis were provided by 10.13039/501100000943Commonwealth Scientific and Industrial Research Organisation (CSIRO). Md. Monirul Islam reports a relationship with 10.13039/501100000943CSIRO that includes: employment, funding grants, non-financial support, and travel reimbursement. If there are other authors, they declare that they have no known competing financial interests or personal relationships that could have appeared to influence the work reported in this paper.
